# Personality Traits and Health Behaviors as Predictors of Fall Among Community-Dwelling Older Adults: Findings From the Canadian Longitudinal Study on Aging

**DOI:** 10.1177/07334648251328427

**Published:** 2025-03-24

**Authors:** Henrietha C. Adandom, Chiedozie J. Alumona, Israel I. Adandom, Adesola C. Odole, Lisa L. Cook, Gongbing Shan, Oluwagbohunmi A. Awosoga

**Affiliations:** 1 Faculty of Health Sciences, 4512 University of Lethbridge, Lethbridge, AB, Canada; 2 Department of Kinesiology, College of Education, 8063 The University of Alabama, Tuscaloosa, AL, USA; 3 Department of Physiotherapy, College of Medicine, 113092 University of Ibadan, Ibadan, Nigeria; 4 Applied Research and Evaluation Services, 3146 Alberta Health Services, Edmonton, AB, Canada; 5 Department of Kinesiology & Physical Education, 4512 University of Lethbridge, Lethbridge, AB, Canada

**Keywords:** personality, health behaviors, falls, older adults, CLSA

## Abstract

**Objectives:** To examine whether personality traits and health behaviors predict falls in community-dwelling older adults. **Methods:** Longitudinal data from the Canadian Longitudinal Study on Aging (CLSA) at baseline (2011–2015) and follow-up two (2018–2021) were analyzed using logistic regression for 5270 adults aged 65 and older, with an alpha level of 0.05. **Results:** At baseline, participants’ mean age was 72 years, with 51.1% female. Most identified as White (96.7%) and had education beyond secondary (81.5%). Increased physical activity (OR: 1.012, 95% CI: 1.01–1.014), decreased alcohol consumption (OR: 1.634, 95% CI: 1.419–1.883), and smoking cessation (OR: 2.8, 95% CI: 2.198–3.568) increased fall risk, while conscientiousness (OR: 0.832, 95% CI: 0.792–0.874) and openness (OR: 0.959, 95% CI: 0.922–0.998) were protective at follow-up two. Personality changes significantly influence falls. **Discussion:** Findings highlight the complex interplay between personality traits, health behaviors, and falls, suggesting a one-size-fits-all approach to fall prevention may be insufficient.

**What this paper adds**

• Findings highlight the complex relationship between smoking behaviors and falls. Notably, smoking cessation in later life significantly increases the likelihood of falls, possibly due to underlying health conditions.
• Moderate drinking demonstrates a protective effect against falls, whereas decreasing drinking or increasing physical activity slightly over time may elevate fall risk in older adults.
• Personality traits such as extraversion, agreeableness, and conscientiousness, along with their changes over time, significantly influence fall risk, providing fresh insights into the psychosocial determinants of physical health in aging populations.

**Applications of study findings**

• Gerontological practitioners can leverage these findings to develop personalized fall prevention strategies, considering both personality traits and changes in health behaviors over time.
• Policies targeting fall prevention in older adults should include education on the potential benefits of moderate alcohol consumption and the risks associated with abrupt changes in physical activity or health behaviors.
• Researchers should explore personality-driven behavior interactions and their longitudinal changes to enhance understanding and development of effective fall prevention interventions for aging populations.

## Introduction

Falls remain a pressing public health issue among older Canadians, with approximately one in three individuals over the age of 65 experiencing a fall each year (Parachute, 2022). The consequences are significant, leading to an estimated $5.6 billion annually in medical expenses associated with both fatal and non-fatal falls (Public Health Agency of Canada, 2022). These incidents not only contribute to loss of functional independence but also impose a substantial burden on healthcare systems and the economy (Florence et al., 2018; Parachute, 2022). Thus, understanding falls factors is critical for prevention and mitigation efforts.

The risk of falls is multifactorial, involving a combination of biological, environmental, and psychological contributors. Biological factors, such as muscle weakness, mobility issues, neurological impairments, and chronic conditions, play a significant role in increasing fall risk, alongside environmental hazards (Lee, 2021; Mielenz et al., 2017). Psychological factors, including depression and cognitive impairment, also substantially contribute to the likelihood of falls. Notably, depression and cognitive impairment often co-occur, compounding the overall risk (Iaboni & Flint, 2013; Stubbs et al., 2016). For example, aging-related declines in multitasking abilities can lead to confusion and attentional conflicts. Depression may exacerbate these cognitive challenges, further reducing an individual’s ability to prevent falls (Fischer et al., 2014; Iaboni & Flint, 2013). While research has extensively explored the biological and environmental contributors to fall risk, the complex interplay between health behaviors, personality traits, and falls remains underexplored.

According to the Health Belief Model (HBM), an adult’s perception of falls—such as perceived susceptibility and severity—and their beliefs about the benefits and barriers to engaging in preventive health behaviors, such as physical activity, may influence fall prevention efforts (Vincenzo et al., 2022). Personality traits can shape these perceptions and the likelihood of engaging in health-promoting behaviors and outcomes, as they are relatively stable characteristics that influence how individuals perceive, interact with, and respond to their environment (Milad & Bogg, 2020; Sanchez-Roige et al., 2018). While this stability may provide a consistent foundation for behavioral patterns, decision making, and coping strategies, individual differences in personality are not perfectly stable overtime, as some persons may experience major live events like a fall, leading to small changes across the lifespan (Bühler et al., 2024; Harris et al., 2016).

The relationship between personality traits and falls has been further explored in research. Canada et al. (2020) conducted a longitudinal cohort study on older adults aged 65–99 years to investigate the relationship between the “big five” personality traits and fall risk. The study found that changes in personality traits, particularly neuroticism and conscientiousness, are significant predictors of falls over time (Canada et al., 2020). Neuroticism, also known as low emotional stability or negative emotions, involves levels of anxiety and emotional instability. A lack of emotional stability may increase fear of falling and heighten falls (Kloseck et al., 2008; Mann et al., 2006). In contrast, extraversion, associated with sociability and assertiveness, has been linked to increased engagement in physical activities, which can elevate the risk of falling (Kloseck et al., 2007, 2008). Conscientiousness, characterized by responsibility and self-discipline, generally correlates with cautious behavior, reducing falls (Canada et al., 2020; Rolison et al., 2014). Meanwhile, agreeableness, reflecting cooperativeness, and empathy may predispose individuals to prioritize others’ needs, potentially increasing their susceptibility to falls (Kloseck et al., 2008; Rolison et al., 2014). Lastly, openness, denoting a preference for new experiences, has been associated with high-risk behaviors (Digman, 1990; Rolison et al., 2014).

Health behaviors, such as tobacco use, alcohol consumption, and physical activity or inactivity, are well-documented determinants of health outcomes (Ding et al., 2015; Feldman & Chaudhury, 2008). Smoking is linked to decreased bone density and poorer overall health, which can increase the risk of falls (Thorin et al., 2016). Physical inactivity contributes to muscle weakness and impaired balance, further raising the risk of falling (Smith et al., 2019; Van Gameren et al., 2022). Alcohol consumption impairs motor function and reaction time, adding to the likelihood of falls (Qian et al., 2021). Unlike personality traits, these behaviors are not inherently stable as individuals may start, stop, or modify these behaviors over time due to life events, interventions, or changing circumstances (Chai et al., 2024). Understanding how these modifiable behaviors contribute to falls in older adults is essential for developing personalized fall prevention strategies, as such insights can empower them to adopt safer behaviors and maintain their independence. In this study, we used data from the Canadian Longitudinal Study on Aging (CLSA) to examine the impact of personality traits and health behaviors on falls among community-dwelling older adults. We hypothesized that (i) personality traits (neuroticism, conscientiousness, openness, extraversion, and agreeableness) and health behaviors (physical activity, alcohol consumption, and tobacco use) predict falls and (ii) that changes in these behaviors over time are associated with the likelihood of falls.

## Methods

### Study Design

**Figure 1.**
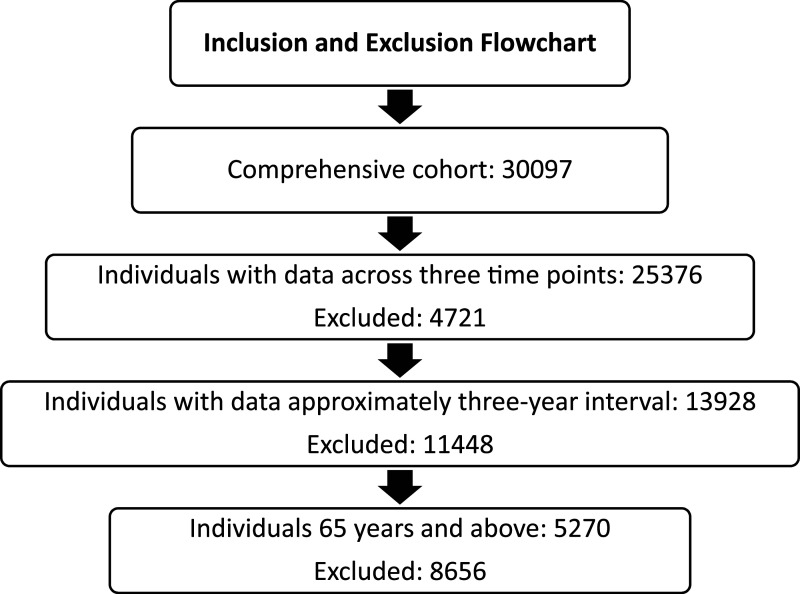
Characteristics of excluded participants.

The study was a secondary longitudinal analysis of the Canadian Longitudinal Study on Aging (CLSA) data at baseline and follow-up two. The CLSA is an ongoing national study that began in 2011, following over 50,000 individuals aged 45–85 at recruitment. Participants were divided into a tracking cohort of 21,241 individuals and a comprehensive cohort of 30,097 individuals (Raina et al., 2009). The CLSA excluded individuals who (1) had cognitive impairment at baseline/recruitment, (2) were full-time members of the Canadian Armed Forces, (3) lived in long-term care facilities at baseline/recruitment and on Federal First Nations reserves or settlements in the Canadian territories, and (4) were unable to communicate in English or French (Raina et al., 2009). We selected 5270 participants from the comprehensive cohort who were 65 years and older at the baseline and completed data collection at baseline (2011–2015), follow-up one (2015–2018), and follow-up two (2018–2021). Figure 1 provides the characteristics of the excluded participants. The study was approved by the Research Ethics Board of University of Alberta (reference number: Pro00129373). For more information about the CLSA, visit https://www.clsa-elcv.ca.

### Outcome Variable

#### Fall Status

Participants were asked to recall falls in the previous year that resulted in limitations to their normal activities using the following question: “In the past 12 months, did you have any falls?” The response options were yes or no. Based on their responses, participants were classified as non-fallers (no) if they had not experienced any falls and fallers (yes) if they had experienced one or more falls.

### Explanatory Variables

#### Personality Traits

The Ten-Item Personality Inventory (TIPI) was used to measure the five personality traits: openness, conscientiousness, extraversion, agreeableness, and emotional stability. The questionnaire was a 7-point Likert scale containing 10 items, with two paired items for each trait. Five of the ten items were reversed-scored, added to their matching pair in the relevant “Big Five” traits, and averaged to provide a single score (range 1 to 7) for each trait (Satia et al., 2022). There was no data for personality traits at follow-up two.

#### Physical Activity

A modified version of the Physical Activity Scale for the Elderly (PASE) was used to measure typical weekly levels of physical activity (Canadian Longitudinal Study on Aging, 2024). The questionnaire asked about the frequency and duration of physical activity in light, moderate, and high-intensity sports, as well as strength training and walking. The response options for the frequency of activity were (0 days), seldom (1 to 2 days), sometimes (3 to 4 days), and often (5 to 7 days), while duration was categorized into less than 30 minutes, 30 minutes to less than one hour, one hour to less than 2 hours, two hours to less than four hours, and four hours or more. The total weekly hours of physical activity were estimated by multiplying the midpoints of the frequency and duration response category except for the ≥4-hour category, which was coded as 4 hours and then summing the results of each activity (Putman et al., 2023). The PASE is a valid and reliable instrument for the assessment of physical activity for use in epidemiology studies in older adults with a test–retest reliability coefficient of 0.75 (Washburn et al., 1993). It also showed good validity when compared with digital technologies like Actigraphy data with a statistically significant Spearman correlation of 0.43 (*p* < .01) (Dinger et al., 2004).

#### Alcohol Consumption

Participants were asked to report their frequency of alcohol consumption over the past 12 months. Response options included almost every day (6–7 times a week), 4–5 times a week, 2–3 times a week, once a week, 2–3 times a month, about once a month, less than once a month, or never. For analysis, participants were categorized as non-drinkers if they reported no alcohol consumption in the past 12 months, regular drinkers if they drank once or more per month, and occasional drinkers if they drank less often than once a month (Canadian Longitudinal Study on Aging, 2021).

#### Smoking History

Participants indicated their smoking history by choosing one of the three responses: yes (I currently smoke), former (I don’t smoke now, but I have in the past), and no (I don’t smoke, and I never have).

#### Covariates

The covariates were sex, age, marital status, education, residence, cultural identity, presence of chronic condition, depression, and cognition. Depression was measured using the Center for Epidemiologic Studies Depression scale (CESD10). It contained 10 items covering depressive symptoms experienced in the past week with four response categories ranging from “zero for rarely” to “four for all of the time.” Scores range from 0 to 30, with <10 or ≥10 indicating negative or positive for depressive symptoms, respectively (Grant et al., 2021). Cognitive function was assessed using two domains from the CLSA: executive function and psychomotor speed. Executive function, measured by the mental alternation test (MAT) and the Stroop test (Victoria version), involves complex behaviors such as planning, decision-making, attention, and problem-solving. Psychomotor speed, assessed by the choice reaction time (CRT) task, involves the coordination of physical movement to react quickly and accurately to external stimuli. Impairments in these cognitive processes can increase falls by compromising the ability to perform daily tasks safely and confidently (Holtzer et al., 2007). Appendix A provides details on the range of scores, measurement methods, and characteristics of each cognitive measure used in the analysis. Z-scores for each cognitive measure were used in this analysis (O’Connell et al., 2022).

#### Variable Description

Continuous variables: age (years), physical activity, depression, Stroop, mental alternation test, choice reaction time, extraversion, agreeableness, conscientiousness, emotional stability, and openness. Categorical variables: fall status (no, yes), sex (female, male), marital status (have partner, do not have a partner), cultural identity (Whites, non-Whites), residence (rural, urban), chronic condition (no, yes), alcohol consumption (non-drinkers, occasional drinkers, regular drinkers), and smoking history (never smoked, current smoker, former smoker). Changes in categorical variables from baseline to follow-up two were categorized as follows: marital status (unchanged/have no partner now, have partner now), smoking history (maintained status, quit smoking, began smoking), alcohol consumption (maintained status, decreased drinking, increased drinking), and chronic condition (no, yes). For all continuous variables, we compared the baseline value to the corresponding value at follow-up two to determine changes in status over time.

### Data Analysis

All analyses were performed using the Statistical Package for Social Sciences (SPSS) version 28, and the alpha level was set at 0.05. According to CLSA guidelines, weights were applied to the dataset. The variables were summarized using descriptive statistics of frequency, percentage, mean, and standard deviation. Prior to inferential statistics, multivariate outliers were assessed using Cook’s distance (Vatcheva et al., 2016). Missing data for the variables were minimal (<5%) and were excluded listwise. Bivariate logistic regression assessed individual associations between each predictor and the outcome, while multivariate logistic regression examined adjusted associations by including all predictors simultaneously, adjusting for others using the forced entry method. Adjusted odds ratios (ORs) and 95% confidence intervals (CIs) were calculated to quantify the strength and direction of associations. Model fit statistics, including −2 Log Likelihood and pseudo-R-squared values (Cox & Snell & Nagelkerke), were reported.

## Results

### Participant’s Characteristics

At baseline, the average participant age was 72 years, with a slightly higher proportion of females (51.1%) than males. The majority were Whites (96.7%) and had an education level above secondary (81.5%). Table 1 shows a significant difference in participants’ fall status between baseline and follow-up two (χ ^2^ = 54.93, *p* < .001), with a high proportion of participants becoming fallers at follow-up two. Significant differences were also observed for smoking history (χ ^2^ = 19.61, *p* < .001) and alcohol consumption (χ ^2^ = 61.66, *p* < .001), with higher proportions of participants currently smoking and being regular drinkers at baseline. There were significant differences in physical activity and personality traits, with a decline in extraversion, agreeableness, emotional stability, and openness at follow-up two (Table 2).

**Table 1.** Participants’ Characteristics.

Variable	Percentage (%)		χ^2^	*p-*Value
	Baseline	Follow up 2		
Marital status			37.413	<0.001*
Do not have a partner	1900 (36.1)	2205 (41.9)		
Have partner	3369 (63.9)	3061(58.1)		
Residence			22.904	<0.001*
Rural	414 (7.9)	290 (5.5)		
Urban	4856 (92.1)	4960 (94.5)		
Smoking history			19.612	<0.001*
Never smoked	2678 (50.8)	2507 (48.7)		
Former smoker (quit)	2356 (44.7)	2478 (48.1)		
Current smoker	235 (4.5)	165 (3.2)		
Alcohol consumption			61.664	<0.001*
Non-drinkers	624 (12.2)	910 (17.3)		
Occasional drinkers	618 (12.0)	683 (13.0)		
Regular drinkers	3892 (75.8)	3668 (69.7)		
Chronic conditions			318.501	<0.001*
No	364 (6.9)	18 (0.7)		
Yes	4886 (93.1)	5132 (99.3)		
Fall status			54.932	<0.001*
No	5002 (94.9)	4808 (91.3)		
Yes	268 (5.1)	461 (8.7)		

*p < .05. χ^2^ = Chi-square statistics.

**Table 2.** Paired Sample T-test for Scale Variables.

Variable	Baseline	Follow-up 2	T-statistic	p-Value
	M ± SD	M ± SD		
Age	72.62 ± 5.52	78.44 ± 5.55	847.180	<0.001*
Physical activity	24.92 ± 24.71	32.20 ± 26.18	21.572	<0.001*
Depression	4.78 ± 4.17	4.79 ± 4.46	0.169	0.866
Stroop	2.25 ± 0.63	2.27 ± 0.84	1.532	0.126
Mental alternation test	25.44 ± 8.45	23.60 ± 7.60	−16.704	<0.001*
Choice reaction time	814.34 ± 190.722	869.544 ± 151.99	11.981	<0.001*
ǂ Extraversion	4.35 ± 1.72	4.21 ± 1.74	−7.431	<0.001*
ǂ Agreeableness	5.94 ± 1.09	5.79 ± 1.14	−9.923	<0.001*
ǂ Conscientiousness	6.21 ± 1.10	6.22± 1.05	0.387	0.699
ǂ Emotional stability	5.93 ± 1.29	5.78 ± 1.31	−9.127	<0.001*
ǂ Openness	5.36 ± 1.40	5.23 ± 1.4	−7.056	<0.001*

*= *p* < .05. ǂ = Analysis was completed with values at baseline and follow-up one.

### Bivariate Analysis

Personality traits and health behaviors demonstrated significant associations with falls at baseline and follow-up assessments (Table 3). At baseline, having no history of smoking (OR: 1.192, 95% CI: 1.112–1.277) and being a regular drinker (OR: 1.215, 95% CI: 1.121–1.317) were significantly associated with higher odds of experiencing falls, while being a current smoker (OR: 0.651, 95% CI: 0.542–0.784), former smoker (OR: 0.899, 95% CI: 0.839–0.964), and occasional drinker (OR: 0.747, 95% CI: 0.666–0.838) were linked to lower odds of falling. Higher levels of all personality traits (extraversion: OR: 1.133, 95% CI: 1.110–1.157; agreeableness: OR: 1.116, 95% CI: 1.079–1.154; conscientiousness: OR: 1.071, 95% CI: 1.035–1.107; emotional stability: OR: 1.061, 95% CI: 1.032–1.091; openness: OR: 1.173, 95% CI: 1.143–1.204) were significantly associated with higher odds of falling.

**Table 3.** Bivariate Binary Logistic Regression for the Association Between Fall Status and Predictors at Baseline and Follow-Up 2.

Variable	Baseline				Follow-up 2			
			95% C.I.				95% C.I.	
	*p*-Value	Odds ratio	Lower	Upper	*p*-Value	Odds ratio	Lower	Upper
Smoking history								
Never smoked	<0.001*	1.192	1.112	1.277	<0.001*	0.753	0.718	0.789
Current smoker	<0.001*	0.651	0.542	0.784	<0.001*	0.392	0.325	0.473
Former smoker (quit)	0.003*	0.899	0.839	0.964	<0.001*	1.448	1.381	1.517
Alcohol consumption								
Non-drinkers	0.181	0.935	0.847	1.032	<0.001*	1.322	1.251	1.397
Occasional drinkers	<0.001*	0.747	0.666	0.838	0.468	1.025	0.959	1.094
Regular drinkers	<0.001*	1.215	1.121	1.317	<0.001*	0.795	0.758	0.834
Physical activity (unit increase)	0.359	1.001	0.999	1.002	<0.001*	0.996	0.995	0.997
ǂ Extraversion (unit increase)	<0.001*	1.133	1.110	1.157	<0.001*	1.113	1.098	1.129
ǂ Agreeableness (unit increase)	<0.001*	1.116	1.079	1.154	<0.001*	0.915	0.897	0.934
ǂ Conscientiousness (unit increase)	<0.001*	1.071	1.035	1.107	<0.001*	0.906	0.887	0.926
ǂ Emotional stability (unit increase)	<0.001*	1.061	1.032	1.091	<0.001*	0.920	0.905	0.935
ǂ Openness (unit increase)	<0.001*	1.173	1.143	1.204	<0.001*	1.120	1.101	1.140

*p < .05. ǂ = Analysis was completed with values at baseline and follow-up one.

In follow-up two, being a former smoker (OR: 1.448, 95% CI: 1.381–1.517), being a non-drinker (OR: 1.322, 95% CI: 1.251–1.397), higher extraversion levels (OR: 1.113, 95% CI: 1.098–1.129), and higher openness levels (OR: 1.120, 95% CI: 1.101–1.140) were significantly associated with higher odds of falling. Conversely, being a non-smoker (OR: 0.753, 95% CI: 0.718–0.789), current smoker (OR: 0.392, 95% CI: 0.325–0.473), and regular drinker (OR: 0.795, 95% CI: 0.758–0.834), and higher levels of physical activity (OR: 0.996, 95% CI: 0.995–0.997), agreeableness (OR: 0.915, 95% CI: 0.897–0.934), conscientiousness (OR: 0.906, 95% CI: 0.887–0.926), and emotional stability (OR: 0.920, 95% CI: 0.905–0.935) were linked to lower odds of falling.

### Multivariate Analysis

Our multivariate analysis revealed complex relationships between falls, personality traits, and health behaviors over time (Table 4). Participants who smoked at baseline (OR: 1.639, 95% CI: 1.171–2.295) had a 63.9% higher likelihood of falling at follow-up two than those who never smoked. Compared to those who maintained their smoking history from baseline, participants who either quit (OR: 2.8, 95% CI: 2.198–3.568) or began smoking (OR: 1.936, 95% CI: 1.291–2.903) by follow-up two had increased odds of falling, with those who quit experiencing the highest increase. Regarding alcohol consumption, compared to non-drinkers at baseline, occasional drinkers were 67.9% less likely to fall at follow-up two (OR: 0.321, 95% CI: 0.268–0.385), and regular drinkers were 61.4% less likely to fall (OR: 0.386, 95% CI: 0.335–0.444). However, participants who decreased drinking (OR: 1.634, 95% CI: 1.419–1.883) by follow-up two had a 63.4% higher likelihood of falling than those who maintained their drinking status. Participants with higher physical activity levels at baseline (OR: 1.014, 95% CI: 1.012–1.016) were associated with a slightly (1.4%) higher likelihood of falling at follow-up two. Interestingly, increased physical activity from baseline to follow-up two (OR: 1.012, 95% CI: 1.010–1.014) was associated with higher odds of falling at follow-up two.

**Table 4.** Multivariate Binary Logistic Regression for the Association Between Fall Status and Predictors at Baseline and Follow-Up 2 (FU2).

Variable	Baseline (BL)				Follow-Up 2 (FU2)			
			95% C.I.				95% C.I.	
	*p*-Value	Odds ratio	Lower	Upper	*p*-Value	Odds ratio	Lower	Upper
Baseline characteristics								
Age (increase in years)	<0.001*	1.018	1.01	1.025	0.807	1.001	0.992	1.011
Sex at birth (ref: female)	<0.001*	0.72	0.659	0.787	<0.001*	0.69	0.618	0.769
Education (ref: secondary and below)	0.160	1.062	0.977	1.154	<0.001*	1.817	1.638	2.016
Cultural identity (ref: non-Whites)	0.018*	1.379	1.056	1.802	0.001*	1.798	1.265	2.556
Marital status (ref has no partner)	<0.001*	0.61	0.561	0.663	<0.001*	0.39	0.352	0.433
Residence (ref: rural)	<0.001*	0.44	0.381	0.509	<0.001*	0.473	0.404	0.553
Smoking history (ref: never smoked)								
Current smoker	0.879	0.985	0.811	1.196	0.004*	1.639	1.171	2.295
Former smoker (quit)	0.124	0.939	0.867	1.017	0.519	0.86	0.543	1.361
Alcohol consumption (ref: non-drinkers)								
Occasional drinkers	<0.001*	0.758	0.649	0.885	<0.001*	0.321	0.268	0.385
Regular drinkers	0.514	0.964	0.865	1.075	<0.001*	0.386	0.335	0.444
Chronic conditions (ref: no)	<0.001*	0.627	0.553	0.711	0.053	0.449	0.2	1.011
Physical activity (unit increase)	0.611	1	0.999	1.002	<0.001*	1.014	1.012	1.016
Depression (unit increase)	<0.001*	1.039	1.029	1.048	0.005*	0.979	0.964	0.994
Stroop (unit increase)	0.582	1.016	0.96	1.075	0.165	0.942	0.866	1.025
Mental alternation test (unit increase)	<0.001*	1.015	1.01	1.02	<0.001*	1.016	1.008	1.023
Choice reaction time (unit increase)	0.116	1	1	1	<0.001*	1.001	1	1.001
Extraversion (unit increase)	<0.001*	1.114	1.087	1.141	<0.001*	1.136	1.103	1.17
Agreeableness (unit increase)	<0.001*	0.939	0.906	0.974	<0.001*	1.114	1.053	1.178
Conscientiousness (unit increase)	<0.001*	1.131	1.086	1.176	<0.001*	0.832	0.792	0.874
Emotional stability (unit increase)	<0.001*	1.161	1.119	1.203	0.146	1.037	0.987	1.089
Openness (unit increase)	<0.001*	1.134	1.1	1.168	0.040*	0.959	0.922	0.998
Change in characteristics								
Marital status change (ref: unchanged/have no partner)	-	-	-	-	<0.001*	1.612	1.37	1.897
Smoking history change (ref: unchanged)								
Began smoking	-	-	-	-	0.001	1.936	1.291	2.903
Quit/stopped smoking	-	-	-	-	<0.001*	2.8	2.198	3.568
Alcohol consumption change (ref: unchanged)								
Decreased drinking	-	-	-	-	<0.001*	1.634	1.419	1.883
Increased drinking	-	-	-	-	0.108	1.146	0.97	1.354
Chronic conditions change (ref: no)	-	-	-	-	0.008*	0.327	0.142	0.751
Physical activity change (unit increase)	-	-	-	-	<0.001*	1.012	1.01	1.014
Depression change (unit increase)	-	-	-	-	<0.001*	1.023	1.009	1.037
Stroop change (unit increase)	-	-	-	-	0.037*	1.084	1.005	1.169
Mental alternation test change (unit decrease)	-	-	-	-	0.854	1.001	0.992	1.009
Choice reaction time change (unit increase)	-	-	-	-	<0.001*	1.001	1.001	1.001
ǂ Extraversion change (unit decrease)	-	-	-	-	<0.001*	1.299	1.247	1.352
ǂ Agreeableness change (unit decline)	-	-	-	-	0.686	0.99	0.944	1.039
ǂ Conscientiousness change (unit increase)	-	-	-	-	<0.001*	0.88	0.835	0.927
ǂ Emotional stability change (unit decrease)	-	-	-	-	<0.001*	0.881	0.843	0.922
ǂ Openness change (unit decline)	-	-	-	-	0.523	0.988	0.95	1.026
Model summary	χ^2^ (20) = 843.495. *p* < .001*				χ^2^ (37) = 1851.816. *p* < .001*			
2 Log likelihood	22,809.710				14,933.384			
Cox & Snell & Nagelkerke R square	0.012; 0.042				0.058; 0.139			

BL = Baseline. FU2 = Follow-up two. *p < .05. ǂ = Analysis was completed with change between BL and FU1.

Regarding personality traits, higher levels of extraversion and agreeableness at baseline were associated with a 13.6% (OR: 1.136, 95% CI: 1.103–1.17) and 11.4% (OR: 1.114, 95% CI: 1.053–1.178) higher likelihood of falling, respectively, at follow-up two. Decreases in extraversion from baseline to follow-up two significantly increased the odds of falling by 29.9% (OR: 1.299, 95% CI: 1.247–1.352). Higher levels of conscientiousness and openness at baseline reduced the odds of falling by 16.8% (OR: 0.832, 95% CI: 0.792–0.874) and 4.1% (OR: 0.959, 95% CI: 0.922–0.998), respectively. Changes in conscientiousness (increase) and emotional stability (decrease) from baseline to follow-up two were linked to a 12% (OR: 0.88, 95% CI: 0.835–0.927) and 11.9% (OR: 0.881, 95% CI: 0.843–0.922) reduced likelihood of falling, respectively (see Table 4).

## Discussion

Our study examined the influence of personality traits and health behaviors on falls among community-dwelling older adults using data from the CLSA. Findings support the hypothesis that these behaviors are significant predictors of falls and that changes in these behaviors over time contribute to the likelihood of falling.

Smoking history emerged as a significant predictor, with participants who smoked showing a 63.9% higher likelihood of falling than those who never smoked. This aligns with evidence linking smoking to poorer balance, muscle strength, and bone density (Al-Bashaireh et al., 2018; Guo et al., 2023). Interestingly, individuals who quit smoking by the follow-up period had significantly higher odds of falling (OR = 2.8) than those who maintained their smoking history or began smoking during the same period. This finding suggests that those who quit smoking may experience other health challenges, such as declining physical health or frailty, that elevate fall risk (Cho et al., 2024; Fahey et al., 2023; Faulkner et al., 2009).

Alcohol consumption showed a protective association, with occasional and regular drinkers having 67.9% and 61.4% reduced odds of falling, respectively, compared to non-drinkers. This may reflect the potential health and social benefits of moderate alcohol consumption, such as improved cardiovascular health, mood regulation, and social engagement, which may reduce fall risk (Reas et al., 2016; Zhang et al., 2020). However, the higher fall risk among participants who decreased their drinking by follow-up two may indicate underlying health changes, frailty, or mobility issues, as well as age-related physiological sensitivity to alcohol, which can impair balance and coordination (White et al., 2023).

The association between increased physical activity and a slight 1.4% higher fall risk aligns with research highlighting the dual-edged nature of physical activity in older adults. While physical activity improves balance, strength, and mobility, it also increases exposure to fall hazards due to greater engagement in potentially risky environments or activities (Kwok et al., 2024; Yuan et al., 2022). This modest increase may reflect a transitional phase in which individuals adopting more active lifestyles encounter temporary risks before realizing full functional benefits. Additionally, significant changes in physical activity levels, particularly abrupt increases, may contribute to short-term increases in fall risk.

Personality traits also demonstrated a complex relationship with falls. Higher levels of extraversion and agreeableness were associated with increased fall risk, likely due to behaviors such as greater social engagement or riskier activities that heighten exposure to fall hazards (Fan et al., 2024; Stephan et al., 2014). The 29.9% increase in fall odds associated with decreased extraversion suggests that social withdrawal and reduced physical activity may also contribute to fall risk (Thomas et al., 2022). Conscientiousness, a trait linked to cautious behavior, was protective against falls (Canada et al., 2020). Interestingly, a decrease in emotional stability (higher neuroticism) reduced the odds of falling, contradicting prior findings (Canada et al., 2020; Stephan et al., 2014). This unexpected result may reflect compensatory behaviors, such as increased vigilance or avoidance of hazardous situations, among individuals with higher neuroticism (Weston & Jackson, 2018). Lastly, openness was associated with a modest 4.1% reduction in fall risk, suggesting that exploratory behaviors may have a limited role in fall prevention.

### Strengths and Limitations

The main strength of our paper lies in its large, representative sample from the Canadian Longitudinal Study on Aging (CLSA), enhancing the generalizability of findings on falls among community-dwelling older adults. Its longitudinal design allows for tracking behavioral changes over time, offering dynamic insights into their impact on falls. By examining both psychological factors, such as personality traits, and health behaviors, including smoking, alcohol use, and physical activity, the study provides a holistic understanding of fall risk. Focusing specifically on significant falls—those limiting normal activities—ensures clinically meaningful results, relevant to fall prevention strategies. Robustness is further enhanced by controlling for potential confounders, enabling accurate associations between personality, behaviors, and falls.

However, limitations exist. Self-reported data for falls and health behaviors may introduce biases, potentially affecting measurement accuracy (Althubaiti, 2016). The complexity of interactions between personality traits and behaviors poses challenges to disentangle their influences fully. Additionally, the inability to distinguish single falls from multiple falls limits insights into predictors of recurrent falls, which may differ from single fall predictors (Pérez-Ros et al., 2019). Finally, attrition bias, common in longitudinal studies, may impact results if follow-up participants differ systematically in fall-related characteristics (Gustavson et al., 2012).

## Conclusion

Our study highlighted the multifaceted relationships between personality traits, health behaviors, and falls in older adults aged 65 years and above. While each factor—smoking, alcohol use, physical activity, and personality—individually influences falls, our findings underscore the importance of considering how these factors interact over time. Personality traits, such as extraversion, conscientiousness, emotional stability, and agreeableness, may drive behavioral changes that either increase or mitigate the likelihood of falls, illustrating the complex interplay between psychological and behavioral domains. Our findings suggest that effective fall prevention strategies for older adults should move beyond addressing individual behaviors in isolation. Instead, interventions should adopt a holistic approach that incorporates personality-driven tendencies and potential behavioral adjustments over time.

## Supplemental Material

Supplemental Material - Personality Traits and Health Behaviors as Predictors of Fall Among Community-Dwelling Older Adults: Findings From the Canadian Longitudinal Study on Aging Supplemental Material for Personality Traits and Health Behaviors as Predictors of Fall Among Community-Dwelling Older Adults: Findings From the Canadian Longitudinal Study on Aging by Henrietha C. Adandom, Chiedozie J. Alumona, Israel I. Adandom, Adesola C. Odole, Lisa L. Cook, Gongbing Shan, and Oluwagbohunmi A. Awosoga in Journal of Applied Gerontology

## Data Availability

The data are available from the Canadian Longitudinal Study on Aging (https://www.clsa-elcv.ca/) for researchers who meet the criteria for access to de-identified CLSA data. The datasets used in the present study were Baseline Comprehensive Dataset (version 7.0), Follow-up 1 Comprehensive Dataset (version 4.0), and Follow-up 2 Comprehensive Dataset (version 2.0).
