# Supplementary material for: Personality Traits and Health Behaviors as Predictors of Fall Among Community-Dwelling Older Adults: Findings From the Canadian Longitudinal Study on Aging
Source: J Appl Gerontol. 2025 Mar 24;45(1):75–85. doi: 10.1177/07334648251328427 (PMC12681367; doi:10.1177/07334648251328427)
Supplement: Supplemental Material - Personality Traits and Health Behaviors as Predictors of Fall Among Community-Dwelling Older Adults: Findings From the Canadian Longitudinal Study on Aging [file sj-pdf-1-jag-10.1177_07334648251328427.pdf]

Appendix A. Summary of Scores. Measurement Methods of The Cognitive Measures Used in This Study

Cognitive Measures	Range of Scores	Measurement Methods
Executive function		
MAT	0-52	MAT. recognized as a concise cognitive switching task. evaluates mental flexibility and processing speed by calculating a score based on the number of accurately alternated pairs of alphabets and numbers (e.g.. 1-A. 2-B) completed within a 30-second timeframe. Elevated scores indicate superior performance (Lee et al.. 2022; Teng. 1995).
Stroop Test: interference ratio (colour/dot)	0.05-38.06	Participants were asked to respond to the color of ink on stimulus cards [i.e.. colored dots. common words printed in same colors as dots. and color words printed in non-corresponding colors of ink]. An interference ratio which divided the time required to complete the last card (i.e.. color) by the time required to complete the first card (i.e.. dot). Lower values reflect better performance (Bayard et al.. 2011; Troyer et al.. 2006).
Psychomotor speed		
CRT: mean response time (ms) without outliers	79-9958	The mean reaction time of the participants. recorded in the CLSA dataset. was calculated as the average of the correct response of the test trials and excluded incorrect answers and timeouts. The scores of correct answers excluding incorrect answers and timeouts were used. Lower values reflect better performance (Lee et al.. 2022; Tuokko et al.. 2020).
